# A Scoping Review of Refugee Children’s Health Conditions, Outcomes, and Measures Used in Refugee-Serving Public Health Centres/Clinics in Canada

**DOI:** 10.3390/ijerph23010092

**Published:** 2026-01-09

**Authors:** Augustine Botwe, Nour Armoush, Cheryl Poth, Sophie Yohani, Rebecca Gokiert

**Affiliations:** 1 Faculty of Education, University of Alberta, Edmonton, AB T6G 2R3, Canada; armoush@ualberta.ca (N.A.); cpoth@ualberta.ca (C.P.); sparkins@ualberta.ca (S.Y.); 2 School of Public Health, University of Alberta, Edmonton, AB T6G 1C9, Canada; rgokiert@ualberta.ca

**Keywords:** refugee, children, health, outcomes, conditions, measurement tools, refugee serving, primary health centre, Canada

## Abstract

**Highlights:**

**Public health relevance—How does this work relate to a public health issue?**

- Refugee children experience a disproportionate burden of infectious diseases, nutritional deficiencies, and developmental and mental health conditions. Current literature pays limited attention to social determinants of health, despite strong evidence that these factors significantly influence refugee child health outcomes.
- The lack of standardized, culturally safe measurement approaches for vulnerable populations, especially refugee children, makes their health needs invisible within the healthcare system, contributing to health inequities. Improving early and equitable assessment is essential to reducing preventable morbidity and enhancing long-term health outcomes.

**Public health significance—Why is this work of significance to public health?**

- Improving how refugee children’s health is measured strengthens the capacity of health systems to anticipate needs, plan services, and prevent future disease burdens. Improved measurement has the potential to act as a preventative strategy that supports population health, reduces long-term cost, and promotes equitable developmental trajectories.
- By revealing gaps in current systems, this study identifies opportunities where coordinated action can reduce avoidable disparities in a rapidly growing, vulnerable population.

**Public health implications—What are the key implications or messages for practitioners, policy makers and/or researchers in public health?**

- Standardized, culturally safe measurement approaches, including evidence-based tools informed by the lived experiences of refugee families, consistent data collection protocols, and shared definitions across jurisdictions, have the potential to improve the accuracy of diagnosis, early identification, surveillance, and equitable access to care for refugee children.
- Strengthened and coordinated data systems, both nationally and internationally, are essential to monitor health conditions and outcomes, understand the influence of social determinants, inform evidence-based policy, and design responsive, high-quality services that address the unique needs of refugee children.

**Abstract:**

Refugee-serving primary health centres/clinics (PHCs) provide culturally safe, integrated care for refugee children, yet little is known about how their health conditions and outcomes are assessed. This scoping review examines the current literature on the health conditions and outcomes of refugee children aged 0–5 years and how they are measured in refugee-serving PHCs in Canada. In partnership with the New Canadians Health Centre and guided by Joanna Briggs Institute methodological guidelines, we systematically searched Medline, CINAHL, Scopus, and Embase. Included studies focused on refugee children in Canada and reported health conditions, outcomes, and their measurements within PHCs. Twenty-five studies (2008–2024) met the inclusion criteria, most from Ontario (*n* = 11), followed by Alberta and Saskatchewan (*n* = 4 each). Reported health conditions or outcomes (*n* = 24) spanned the physical (*n* = 19), developmental, and mental health domains (*n* = 5). Communicable (e.g., gastrointestinal infections, hepatitis) and non-communicable conditions (e.g., malnutrition, vitamin D deficiency) were mostly reported. Although some standardized approaches were used, substantial variability exists across provinces and conditions or outcomes measured. Findings reveal a disproportionate focus on physical health and notable variability and gaps in child health measures, limited cultural adaptation, and lack of longitudinal data. Standardized, culturally responsive, and age-appropriate measurement approaches are needed to enhance health equity and inform evidence-based policy for refugee children in Canada.

## 1. Introduction

By the end of 2024, it was estimated that over 123.2 million people were forcibly displaced worldwide [1] due to armed conflict, human rights violations, and climate-driven displacement [1,2]. As global crises intensify, an increasing number of people are forced to flee their homes, further escalating the global emergency. In 2024, just four countries accounted for 94% of global resettlement arrivals: the United States (105,500), Canada (49,300), Australia (17,200), and Germany (5600) [1]. Among countries with universal healthcare coverage, Canada plays a vital role in welcoming asylum claimants and refugees, receiving approximately 200,000 in 2023. While the country provides essential healthcare support to refugees, greater attention is needed to fully address the complex and evolving health needs of this population.

The majority of refugees, specifically, government-assisted refugees (GARs), are eligible for universal healthcare access upon arrival in Canada through Interim Federal Health Program coverage [3]. This coverage includes essential and urgent medical services, immunizations, diagnostic testing, hospital care, prescription medications, and limited dental and vision care until they become eligible for provincial or territorial health insurance. Although GARs and Canadian citizens are eligible for the same healthcare, refugees face unique barriers, such as language, unfamiliarity with the healthcare system, and navigation challenges, which create healthcare access disparities.

The disparity in healthcare access is particularly consequential for refugee children, especially those younger than six years, due to their increased vulnerability, which is often attributed to missed immunizations, vitamin D deficiency, developmental delays, and malnutrition, including stunting, wasting, and underweight [4,5,6]. Given the heightened risks for refugee children, addressing their healthcare needs requires a unique approach to service delivery to mitigate the long-term consequences of unmet healthcare needs.

System-level perspectives and corresponding policies that consider health needs across time and context are required for effective refugee healthcare access and system delivery [7,8,9]. Additionally, being able to clearly define and measure these needs and outcomes is critical for effectively addressing health disparities and informing equitable service delivery. Throughout the literature, several terminologies are used to denote health needs, health conditions, health issues, disorders, and health status and are used interchangeably. For this study, health condition is used as an overarching term to include all of these terminologies and to mean the gap between an individual’s current health status and/or a standard level of health, often shaped by social determinants such as housing, education, and income [10,11,12,13,14]. Health conditions can vary from higher rates of communicable diseases and malnutrition to mental health conditions [15,16,17]. Similarly, health outcome is also used interchangeably in the literature, and for this study, health outcome refers to the results of healthcare interventions or lack thereof, and broader social and environmental influences, which include changes in health status, behaviour, or knowledge due to healthcare or public health efforts [15,16,18,19,20,21]. These two broad terminologies, health conditions and outcomes, allow for a system-level understanding of refugee children’s health that integrates clinical, social, and policy dimensions and reflects the complex interactions between individual health experiences and structural determinants.

Within the Canadian universal healthcare system, refugee-serving primary healthcare centres/clinics (PHCs) play a critical role in delivering care tailored to the unique healthcare conditions and outcomes of refugee children and their families. Examples of refugee-serving PHCs in Canada include the New Canadian Clinic in British Columbia, the Sanctuary Refugee Health Centre in Ontario, Mosaic Refugee Health Clinic and the New Canadian Health Centre (NCHC) in Alberta [17,22]. Refugee-serving PHCs adopt systems-level approaches grounded in three key principles: a holistic view of health, a life-course approach, and culturally safe care [15,16,17,18,19,20,21,22]. The holistic view of health, which is linked with a life-course approach, emphasizes that refugee children’s health is shaped by both clinical conditions and outcomes, and the social determinants of health, including socioeconomic status, language barriers, access to social services, and cultural beliefs about health, as well as their interactions with the healthcare system [11,14,22]. This approach highlights the importance of early intervention during the critical early developmental periods to support optimal development and long-term well-being [16,20,21]. Culturally safe care ensures that healthcare is respectful, equitable, and responsive to the cultural identities and lived experiences of refugee families, while promoting patient empowerment and involvement in decisions about their care [23,24,25,26,27]. Together, these integrated approaches can mitigate systemic barriers and improve healthcare access for refugee children [15,27].

Despite the growing recognition of refugee children’s complex and urgent health conditions and outcomes, most research remains disproportionately focused on adolescents, leaving infants and preschool-aged children underrepresented in the literature [10,11,28,29]. Significant gaps also persist in the measurement of health conditions and outcomes [29,30,31,32]. Furthermore, the measurement of refugee children’s health conditions or outcomes remains underdeveloped in the Canadian literature, with few studies offering systematic approaches [30]. However, the specific approaches, tools, and processes used to measure these conditions, such as screening instruments, diagnostic pathways, assessment protocols, and documentation practices, remain poorly described in the literature. No studies to date have mapped how refugee-serving PHCs operationalize measurement to capture children’s conditions and outcomes.

A recent review by Higgins et al. [33] examined the health conditions of refugee children aged 0–6 years in high-income countries, identifying a high prevalence of infectious diseases, nutritional deficiencies, and developmental delays. While Higgins et al.’s review provides valuable insights into the types of health conditions affecting refugee children, it did not examine how these conditions were identified, the measurement approaches used, or whether PHC settings employed standardized, culturally safe, or developmentally appropriate tools. To the authors’ knowledge, no existing review has focused on the Canadian context or examined measurement practices, specifically within refugee-serving PHCs. This scoping review therefore addresses these critical gaps by examining the health conditions and outcomes of refugee children from birth to five years old and how they are measured in refugee-serving PHCs in Canada. It focuses specifically on the Canadian context and the refugee-serving PHC environment. Through inductive analysis, we categorize the findings by capturing both the types of health conditions and outcomes and measurement approaches, including methodologies, techniques, and tools. This review provides a more targeted analysis of the structural and procedural dimensions of refugee child health measurement in Canada and extends the contributions of Higgins et al. Understanding these measurement approaches is critical, as they influence clinical care, policy decisions, resource allocation, and long-term health planning. Moreover, by systematically mapping the current evidence, this review identifies inconsistencies and gaps, highlights best practices, and proposes approaches to improve the measurement of refugee child health conditions and outcomes. This study is especially timely, given the growing refugee population in Canada and the need for a coordinated, systems-level response that ensures equitable and effective care.

## 2. Materials and Methods

The review followed the methodological guidelines from the Joanna Briggs Institute (JBI) for scoping reviews [34,35] and was reported according to PRISMA extensions for scoping reviews (PRISMA-ScR), designing, conducting, and reporting results [35,36]. This review was conducted in partnership with the New Canadians Health Centre (NCHC), a refugee-serving PHC located in Edmonton, Canada, which has a vested interest in learning about the health conditions and outcomes measured and reported by other refugee-serving PHCs in Canada. The NCHC provides culturally safe healthcare services to government-assisted refugees, and its operations are grounded in the principles of social justice, equity, and inclusion [16]. The centre’s Research and Evaluation Committee (REC), composed of interdisciplinary researchers, healthcare practitioners, and refugees with lived experience, played a central role in guiding this review. The REC was equitably involved in all stages of the review, including the interpretation of the findings, enhancing its relevance and benefit to the community, as well as ensuring the review reflected both academic rigour and community priorities [37,38].

The review protocol was registered in the Open Science Framework (https://doi.org/10.17605/OSF.IO/5C2PN) on 5 November 2024, ensuring transparency and methodological rigour. The data used in this study were all publicly available, and ethics approval was not required. In the section that follows, we outline the key steps of this protocol.

### 2.1. Identifying the Research Question

Through consultation with the REC, three research questions to guide the scoping review were identified: (1) What are the current refugee child health conditions and outcomes described in the literature in Canada? (2) What tools have been reported in the literature to assess these conditions? and (3) What are the methodological challenges and the intersectional considerations for refugee children’s health in Canada?

### 2.2. Identifying Relevant Studies

#### 2.2.1. Search Strategy

Search terms were defined according to the Patient, Intervention Comparison and Outcome (PICO) criteria [39]. Relevant search terms for each aspect of PICO were gathered and applied in our systematic search. A health science librarian assisted in refining our search strategy and in conducting the search. In May 2024, we systematically searched the following databases: EMBASE, Global Health, Medline, CINAHL Plus with full text via Ebscohost, and Scopus Advanced Search for literature published from 1949 to 2024. The wide period ensured an inclusive understanding of the evolving health conditions and outcomes within refugee-serving PHCs in Canada. Slight adaptations of the search string were made for each database’s specific requirements.

We subsequently completed a grey literature search in November 2024. Grey literature was identified through targeted Google Advanced Search, Google Scholar, and relevant refugee and immigrant-serving PHC website searches using the keywords applied in our database search. However, we did not identify any sources that did not meet our eligibility criteria. Further, systematic reference and citation screening was performed on the retrieved full texts. To capture the most recent studies, an updated search was conducted on 17 May 2025.

#### 2.2.2. Eligibility Criteria

Inclusion and exclusion criteria were defined by population, age, setting, phenomena of interest, study design, publication type, country, and language. To be eligible, studies needed to include (i) refugee children 0–5 years old in the population, (ii) child health outcomes or conditions, (iii) health condition or outcome measurement tools, and (iv) primary healthcare centres or clinics providing services to refugees/immigrants. Studies addressing health conditions and outcome measurement tools were included only if they were specific to the target population (i.e., refugee children aged 0–5). Exceptions were made for studies on measurements that explicitly focused on developmental delays or disabilities, mental health, or behaviour-related issues in children, even if the population was broader and covered the general pediatric population (0–18 years). This is because these health conditions and outcome measurements are generally developed for the pediatric population, 0–18 years old. Two independent reviewers (AB and NA) screened the articles using these criteria and refined them through an iterative process. The full final inclusion and exclusion criteria are presented in Table 1 and Box A1 in Appendix A.

### 2.3. Study Selection

All identified studies were imported into Covidence software (2025) [40], a web-based platform for systematic review collaboration, and duplicates were removed automatically. Screening and full-text review were conducted independently and in duplicate by two researchers (AB and NA). Titles and abstracts were screened based on predefined inclusion and exclusion criteria, with an initial agreement rate of 82%. Disagreements were resolved through discussion and consensus. Full-text articles of included studies were then reviewed using the same process. A.B. and N.A. independently assessed each study against the criteria, and any disagreements were again resolved by consensus. Following the full-text review, both researchers independently conducted citation and reference searches of the included studies. These additional records were screened and reviewed using the same procedures described above.

Additional studies were identified through searches of Google Scholar and grey literature. These were screened using the same inclusion criteria and review process described above. However, none of the grey literature sources met the inclusion criteria, and the studies found through Google Scholar were duplicates of those already included.

### 2.4. Data Extraction

A data extraction spreadsheet was created in Covidence, which captured key study details, including publication information (authors, title, and year), study aim, patients’ demographics (age, gender, and country/region of origin), provinces where the study was conducted, study design, health conditions or outcomes, and their measurement (where applicable). After piloting the spreadsheet on a few articles, it was refined to include additional fields such as data sources, type of refugee or immigrant population, detailed descriptions of outcome measurements, key findings, and discussion points (see Table A1).

Two researchers (AB and NA) independently extracted the data into Covidence. Each entry was cross-checked by the other researcher to ensure accuracy before finalization. The countries of origin for study participants were categorized using the World Health Organization’s regional groupings.

### 2.5. Synthesis of Extracted Results

The extracted data were synthesized and organized according to the study’s research questions. A descriptive summary of study characteristics was first developed, followed by a thematic analysis focused on child health conditions and outcomes, and how they are measured. The analysis began with familiarization with the data, followed by inductive coding of text related to health conditions, outcomes, and measurement approaches. These codes were grouped into broader themes that reflected recurring patterns across studies. Themes were refined through iterative comparison and validation between researchers (A.B. and N.A.) to ensure consistency and relevance. The final themes captured key insights into the types of child health conditions and outcomes reported, such as developmental and mental health (e.g., behavioural difficulties, ADHD, emotional dysregulation, aggression, anxiety, and depression), as well as physical health. The methods used to assess these outcomes included clinical assessments, parental reports, standardized tools, and consideration of contextual factors influencing health.

## 3. Results

The results are presented in two sections: Section 1 describes the demographics of study characteristics, including patients’ age, gender, country or region of origin, and the health conditions and outcomes identified across the studies. Section 2 presents the thematic analysis, identifying themes on physical, developmental, and mental health conditions or outcomes and measurements. Section 2 is organized by research questions.

### 3.1. Studies Included

**Figure 1:**
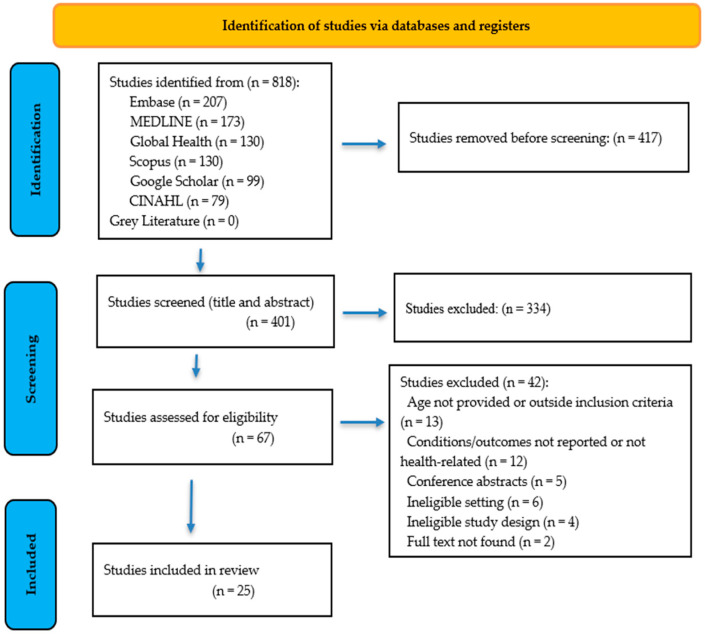
Preferred Reporting Items for Systematic reviews and Meta-Analyses extension for Scoping Reviews (PRISMA-ScR) flow diagram.

A total of 818 articles, including Google Scholar articles, were retrieved based on our systematic search strategy. After removing duplicates, title and abstract screening, and full-text review, a total of 25 articles were included in the review. See Figure 1 for the PRISMA flow diagram. A completed PRISMA checklist is provided in the Supplementary Material (File S1) accompanying this manuscript to document compliance with standardized reporting guidelines for systematic reviews.

### 3.2. Study Characteristics

The studies were published between 2008 and 2024, with nearly half published within the last five years. See Table A1 and Table A2, which present summaries of studies included, study aim, their design, population characteristics, health conditions and outcome categories, and their measurement. Most studies were conducted in Ontario (*n* = 11), followed by Alberta and Saskatchewan (*n* = 4 each), Quebec (*n* = 2), British Columbia (*n* = 1), national-level studies (*n* = 2), and one study spanning Ontario and Quebec, all provinces in Canada. Twenty-two of the studies were cross-sectional studies, and three were cohort studies. Of 25 studies, 20 employed quantitative methods, four used mixed methods, and one was a qualitative study. Most of the studies were retrospective designs (*n* = 24), with only one prospective study. A total of 21 of the 25 included studies presented information on health conditions or outcome measurements (Table A1). Electronic medical record (EMR) chart reviews were the predominant data source for assessing health conditions or outcomes (*n* = 15), followed by surveys (*n* = 3), health screenings (*n* = 3), administrative databases (*n* = 2), and clinical assessments (*n* = 2).

Thirteen studies reported disaggregated outcomes for children aged 0–5, while 19 included children older than six. Gender was reported in 22 of the 25 studies, with female representation ranging from 40% to 57% (mean = 49%, SD = 5.0). Two studies included refugees and claimants, one included refugees and asylum seekers, and the remaining 22 focused solely on refugees. Half of the studies (*n* = 12) did not specify refugee categories. Among the 13 that did, eight involved Government-Assisted Refugees (GARs), four included both GARs and Privately Sponsored Refugees (PSRs), and one included GARs, PSRs, and Blended Visa Office-Referred Refugees. Twenty studies reported participants’ countries of origin, representing 28 countries, primarily from the Middle East, Asia, and sub-Saharan Africa.

### 3.3. Refugee Child Health Conditions and Outcomes in Canada

A thematic analysis of extracted data found 24 distinct health conditions and outcomes across the 25 included studies. Although many studies have reported conditions and outcomes at various age ranges, we specifically focus on those relevant to children from birth to five years. The 24 health conditions and outcomes were further categorized into physical health (*n* = 20), developmental, and mental health (*n* = 5). Of the 20 studies on physical health, 21 unique health conditions and outcomes were identified and categorized into communicable and non-communicable diseases/conditions. Table 2 below, presents a summary of results at a glance.

#### 3.3.1. Communicable Diseases Conditions and Outcomes

The most commonly reported communicable diseases included gastrointestinal infections (*n* = 6) [33,41,42,43,44,45], parasitic infections (*n* = 5) [33,41,46,47,48], and hepatitis (*n* = 4) [33,41,43,47]. Gastrointestinal infections, including Giardiasis and Cryptosporidiosis, and parasitic infections, including worms, Strongyloides stercoralis, and Schistosoma species, were often interrelated, and typically a result of contaminated food and water, conditions frequently encountered in refugee camps or during migration journeys. Hepatitis, particularly type B, is likely due to a lack of or missed childhood immunizations [49]. Three studies [33,48,50] reported on tuberculosis or latent tuberculosis infections (LTBIs).

#### 3.3.2. Non-Communicable Diseases Conditions and Outcomes

A total of 19 different non-communicable diseases/conditions were identified across the included studies [33,41,43,44,45,46,47,48,51,52,53,54,55,56,57,58,59,60,61]. The most commonly reported were malnutrition and bone density (*n* = 10) [33,41,46,49,52,53,54,56,57], which often co-occurred. Malnutrition included both undernutrition and micronutrient deficiencies, often linked to food insecurity and inadequate dietary intake. Bone health conditions, such as rickets, were associated with insufficient vitamin D and calcium levels. Haematological conditions, including anemia (*n* = 5) [33,41,46,48,60], were also prevalent. While anaemia [33,41,46,48,60] and iron deficiency [33,48] were sometimes reported separately, anaemia in children was attributed to chronic illness and genetic conditions. Immunization status (*n* = 4) [33,47,49,52], with incomplete, delayed, or no immunization, was identified as a contributing factor to the persistence of preventable infectious diseases. Oral health conditions (*n* = 4) [33,57,58,59] included dental caries and periodontal disease, frequently linked to poor oral hygiene practices and limited access to dental care services. Lastly, vitamin deficiencies (*n* = 4) [33,43,55,61], particularly vitamin A, D, and B complex, were associated with a range of health conditions, including impaired immunity, vision problems, and neurological symptoms.

#### 3.3.3. Developmental and Mental Health of Refugee Children

A total of five studies [33,51,55,56,60] reported on the developmental and mental health of refugee children. These health conditions focused primarily on neurodevelopmental delays and disabilities, behavioural difficulties including attention-deficit/hyperactivity disorder (ADHD) and emotion dysregulation, aggression, anxiety, and depression. These studies described the effects of child developmental and mental health conditions through neurodevelopmental delays and disabilities, behavioural and emotional dysregulation, such as irritability, excessive or aggressive behaviours, tantrums, social withdrawal, and loss of interest in daily activities or difficulty with attention. This complex interplay of these conditions and their effects on child developmental outcomes was also noted.

### 3.4. Measurement of Refugee Child Health Conditions and Outcomes by Refugee-Serving PHCs

Using the overarching categories of communicable and non-communicable diseases and developmental and mental health, the specificity of how health conditions and outcomes are measured varied considerably, as detailed below. Generally, most articles did not specify the age range that was appropriate for each tool. Therefore, it is assumed that measurement approaches, including methodologies, techniques, and tools, are used for children five years or younger, unless otherwise stated.

Among the studies included, only one study [56] provided the measurement approach for all three categories: communicable, non-communicable, and developmental and mental health. The study authors utilized electronic medical records as a secondary data source, and reviewers manually diagnosed clinical conditions and outcomes per the International Classification of Diseases and Related Health Problems, the 10th Revision, Canada (ICD-10-CA).

#### 3.4.1. Measures of Communicable Diseases

Six studies [41,42,45,46,48,50] reported on the approaches used to measure various communicable diseases, including intestinal parasites and respiratory infections. All studies that reported on their parasite detection method used the stool ova and parasites (O&P) test [41,42,46,48]. Specific approaches varied across laboratories. In one study [45], conducted in Quebec, Canada, positive microscopy results were used for initial detection, followed by enzyme immunoassay to differentiate Entamoeba species, whereas in another study [42], conducted in Alberta, Canada, enzyme immunoassay was used initially to screen for *Giardia* and *Cryptosporidium*, followed by microscopic examination of all specimens. While both studies employed microscopy and enzyme immunoassays, the order and purpose of the tools varied.

In addition to approach variations, some studies have differentiated between active infections and prior exposure. In a study [41] conducted in Ontario, hepatitis B surface antigen (HBsAg) was tested to detect active infection and for hepatitis B core antibody (anti-HBc) to assess prior exposure. Prior exposure to varicella was measured through an immunoglobulin G (IgG) positive test. Lastly, a study conducted in Saskatchewan, Canada [46] provided information on measuring latent tuberculosis infection through two different methods: Interferon-Gamma Release Assay (IGRA) and Tuberculin Skin Test. The Interferon-Gamma Release Assay was used for children older than two years, while the Tuberculin Skin Test was used for children between 6 months and 2 years.

#### 3.4.2. Measures of Non-Communicable Diseases

##### (1) Growth/Malnutrition/Bone Density

Ten studies [41,43,46,48,49,52,54,55,57,61] reported on the measurement approaches used to assess children’s growth. Although a variety of measures were used, the most common was a simple measurement of height and weight to determine body mass index, based on the World Health Organization’s (WHO) criteria. One study [48] used both the Centers for Disease Control and Prevention Clinical Growth Charts and the WHO Child Growth Standards to norm-reference height and weight. Comparatively, Guttman [52] categorized birth weight by predetermined criteria: very low (400–1499 g), low (1500–2499 g), and normal (32,500 g). Another study [57] used the dual-energy X-ray absorptiometry (DXA) machine, which measured body composition, bone mineral content, and density of total body, hip, and lumbar spine. Although all children (3–13) underwent the DXA scan, clinical reference standards were not available for children younger than 8 years. Weight was interpreted using the American anthropometric standards for children aged 5–13, and DXA-specific standards for those aged 8–13. Waist circumference was measured using the American standards for children 5 years and older, and the Canadian standards were applied for those aged 11–13 years.

Across the six studies [43,52,55,57,61,62] that measured vitamin D, most measured serum 25-hydroxyvitamin D (25[OH]D), the primary circulating form of vitamin D. However, interpretations of serum levels varied, with some studies referencing the Institute of Medicine, Osteoporosis Canada, or the Canadian Pediatric Society guidelines. There was no consensus on the threshold for sufficiency, with some studies using 50 nmol/L and others 75 nmol/L as the cutoff. In one study [60], in addition to serum measurement, researchers also administered the Vitamin D Food Frequency Questionnaire [63], which collected data on the type and frequency of foods and drinks consumed. Another study [54] indirectly measured vitamin D status by reporting whether infants received vitamin D supplements, as recommended by Health Canada [62], for infants and young children.

##### (2) Anaemia/Haematological Conditions

Anemia was measured in three studies [46,48,57] using hemoglobin levels. Two studies [48,57] followed the WHO threshold of less than 110 g/L for children aged 6–59 months, while the third study [46] used unspecified age-specific laboratory criteria.

##### (3) Immunization Status

Immunization status was measured in two papers [49,52]. One of the two studies [52] categorized immunization rates by the percentage of complete immunization but did not define the exact vaccines required. Another study [49] defined complete immunization as three doses of Diphtheria/Pertussis/Tetanus/Polio/Haemophilus influenzae type B (DPTP/Hib) with a booster and one dose of measles, mumps, and rubella vaccine.

##### (4) Diabetes

Three studies [41,57,62] measured diabetes by measuring glucose levels. One of the three studies [62] measured serum glucose using a random capillary blood glucose test and classified levels ≥ 7.8 mmol/L as high. Another study [41] followed the 2013 Canadian Diabetes Association guidelines, identifying prediabetes or diabetes based on fasting glucose ≥ 6.1 mmol/L, random glucose ≥ 11.1 mmol/L, or HbA1c ≥ 6.0%. Similarly, one of the studies [57] used fasting glucose, random glucose, and HbA1c to define impaired glucose metabolism, with thresholds of ≥7.0 mmol/L, ≥11.1 mmol/L, and ≥6.5%, respectively.

##### (5) Oral Health

Two studies [58,59] measured children’s oral health through clinical examinations. Clinical examinations were used to measure multiple oral health indicators, including dental caries, oral hygiene, gingival health, and malocclusion status. Dental caries was diagnosed according to the WHO standard criteria, which counted the number of decayed, missing, and filled primary and permanent teeth (dmft/DMFT). Oral hygiene was measured based on the amount of plaque accumulation and quantified using the Simplified Oral Hygiene Index. Gingivitis and malocclusion were both measured through visual inspection, based on signs of redness, swelling, and spontaneous bleeding, and the appearance of cross-bite, open bite, overbite, and overjet. Additionally, urgent treatment needs were recorded for pain and infection, extractions, restorations, orthodontics, plaque control instructions, scaling, and root planning.

##### (6) Eye Health

Only one study [51] reported eye health, specifically ocular health status. Data collection included self-reported medical history, including ocular histories, clinical assessments, and interviews assessing subjective visual acuity and access to eye care. The clinical assessments involved visual screening, slit-lamp examinations, direct dilated fundoscopy, and refractive index measurements.

#### 3.4.3. Developmental and Mental Health

Overall, six studies [33,45,52,54,60,64] reported on developmental and mental health conditions with varying foci and measurement tools. Three studies [52,54,60] reported information on the measurement of child development. One study [45] in Ontario, Canada, reported on the uptake of the enhanced 18-month well-child visit (EWCV), a developmental check-up to evaluate expected milestones. In the two other studies, several measures were used to assess developmental disabilities and delays, including the Nipissing District Assessment tool; the Rourke Baby Record; the Parents Evaluation of Developmental Status; Ages and Stages Questionnaire; the Autism Diagnostic Observation Schedule; the Modified Checklist for autism in toddlers; and the Childhood Autism Rating Scale screening tools. These tools are recommended by both the Canadian Pediatric Society and the American Academy of Pediatrics. The current standard of care for children includes Surveillance and Standardized Developmental and Behavioural Screening [31,65,66,67,68,69]. The Nipissing District Assessment Tool, a standardized developmental screening tool, was used to monitor the developmental progress of children from one month to six years of age for early intervention. The tool includes a series of age-based questionnaires (e.g., 2 months, 6 months, 12 months, etc.) that assess a child’s development across key domains. This tool is widely used in Canada and internationally by health professionals, early childhood educators, and parents. The Rourke Baby Record is used for children from birth to five years and provides guidelines on developmentally appropriate milestones [66,67,68,69,70]. Similarly, the Parents’ Evaluation of Developmental Status and the Ages and Stages Questionnaire are used for children from birth to 11 years and four months to five years, respectively. Several tools are used for the screening and measurement of autism spectrum disorder (ASD); however, the Autism Diagnostic Observation Schedule, the Modified Checklist for Autism in Toddlers, and the Childhood Autism Rating Scale Questionnaire screening tools is preferred.

All the studies [33,45,52,60,64] that measured mental health conditions and outcomes utilized different measurement tools based on the availability of local data. One study [64] leveraged the 2014 Ontario Child Health Study Emotional Behavioural Scales, which is a parent/caregiver-report tool that evaluates internalizing and externalizing symptoms to measure seven mental health disorders (using the fifth edition of the Diagnostic and Statistical Manual of Mental Disorders-5). Internalizing behaviours include measuring symptoms of generalized anxiety disorder, separation anxiety disorder, major depressive disorder, and social phobia/social anxiety disorder, while externalizing behaviours include measuring symptoms of attention deficit hyperactivity disorder (ADHD), oppositional defiant disorder, and conduct disorder. Another study [71] relied on the use of multiple databases to investigate cases of ADHD, conduct disorders, and mood/anxiety disorders among immigrant, refugee, and nonimmigrant children in the province of British Columbia, Canada. The authors based their indicators on another Canadian provincial health policy, the Manitoba Centre for Health Policy’s operational definitions, which were developed using International Classification of Diseases (ICD-9 and ICD-10) codes. Diagnoses were determined through a combination of drug dispensation and diagnoses of several different types of disorders. For instance, children diagnosed with hyperkinetic syndrome and prescribed ADHD medication were classified as having ADHD.

## 4. Discussion

This section first discusses the key findings describing health conditions and outcomes in the current literature and then examines the measurement approaches for the conditions and outcomes. Next, the methodological and contextual challenges in measurement practices are discussed, with considerations of intersectionality, followed by an exploration of the implications of the review findings for research, clinical practice, and policy. To conclude, recommendations to enhance the measurement and delivery of refugee child health services in Canada are proposed.

The findings from this review are situated within the broader context of health equity and culturally safe care. Refugee children’s health is not solely determined by a clinical condition, but by the structures and systems that shape access to prevention, diagnosis, and follow-up care to support the achievement of their outcomes. Measurement practices therefore reflect assumptions, cultural norms, and institutional priorities that can either mitigate or amplify inequities. Across the studies reviewed, disparities in how conditions and outcomes are recorded reveal systemic inequities that disproportionately affect young refugee children. By examining not only what health conditions exist but how they are made visible through measurement, this review highlights measurements as a determinant of health equity.

### 4.1. Refugee Child Health Conditions and Outcomes

The findings from this review align with global data but also bring to the fore unique challenges in the Canadian healthcare and research context. Many studies aggregate data across broad pediatric age ranges (0–18 years) or focus specifically on adolescents (13–18 years), which obscures the unique developmental and health barriers faced by younger refugee children, limiting their ability to generate age-specific insights and interventions. Communicable diseases, particularly hepatitis A and B, remain a significant concern. Globally, hepatitis B alone causes approximately 800,000 deaths annually for all age groups [72], and recent studies confirm its continued burden in vulnerable populations [73]. The review findings align with the global trend, indicating that viral hepatitis remains a major contributor to global morbidity and mortality, particularly in regions with limited access to vaccination and treatment programs [28,73]. In Canada, refugee children are at heightened risk due to pre-migration exposures and post-migration conditions such as overcrowded shelters and poor sanitation [28,50,56]. While Canada has strong vaccination programs, the re-emergence of vaccine-preventable diseases suggests gaps in immunization coverage, follow-up care, and surveillance, not just among refugee populations but among the general population. This calls for enhanced early screening, catch-up vaccination, and monitoring to prevent outbreaks.

Non-communicable diseases are also prevalent. These often co-occur, shaped by both pre- and post-migration factors. While these findings are consistent with broader Canadian child health data, refugee children face compounded risks due to systemic barriers to accessing care [10,17,28]. The literature supports the need for nutrition-sensitive, integrated, culturally safe healthcare approaches, yet evidence on long-term outcomes and the effectiveness of intervention remains limited, indicating a critical area for future research.

Despite growing recognition of the psychological toll of forced migration, few Canadian studies focus specifically on young refugee children’s developmental and mental health. This gap is consistent with findings from Higgins et al. [33] and supported by findings from the Centre for Addiction and Mental Health and the Canadian Medical Association Journal (CMAJ) [6]. The co-occurrence of anxiety and depression combined with the diagnostic challenges in assessing young children and cultural stigma associated with mental health may have contributed to the underreporting and underdiagnosis [74,75]. While tools like the CMAJ-endorsed screening checklist [76,77] offer guidance, Bhayana & Bhayana [60] emphasize the need to integrate these tools into broader clinical guidelines from the Canadian Pediatric Society and the Centre for Disease Control (CDC) to ensure trauma-informed, culturally safe care.

This review confirms what is already known globally: refugee children are at risk for both infectious and chronic conditions. However, in the Canadian context, the literature remains fragmented, with limited longitudinal data, inconsistent reporting on developmental outcomes, and a lack of studies on tropical or parasitic diseases that may be unfamiliar to Canadian clinicians. This raises concerns about the preparedness of the healthcare system to manage conditions that are rare in the general population but increasingly relevant due to global migration and global climate change. Addressing these challenges requires Canada to strengthen its early screening programs, ensure equitable access to vaccinations, expand culturally safe care, particularly for mental health services, and invest in longitudinal research that captures the evolving health conditions of young refugee children.

### 4.2. Measurement Inconsistencies and Their Implications for Refugee Child Health Assessment

This review highlights the lack of age-specific details and raises questions about the appropriateness and accuracy of some measurement approaches for young children, particularly in the context of refugee health, where developmental and cultural considerations are critical, yet often overlooked. Additionally, this study identified heterogeneity in the measurement of health conditions and outcomes among refugee children in Canada. While some measurement practices align with established clinical guidelines, others differ widely between provinces and institutions, reflecting broader systemic inconsistencies in pediatric health assessment. These inconsistencies stem from a lack of standardization in measurement techniques and tools, protocols, and reference criteria. These findings raise critical questions about the reliability, comparability, and clinical utility of current practices. This is especially concerning in refugee health, where early and accurate measurement is essential, and the preparedness of the Canadian healthcare system is vital.

For communicable diseases, while some measurement techniques and tools, such as those used to detect latent tuberculosis infection, are standardized across Canada, others show significant variations. For example, the detection of stool ova and intestinal parasites differs notably between provinces due to variations in public health guidelines, laboratory infrastructure and protocols, and test availability. These differences are driven more by provincial systems than by refugee-specific considerations. In Müller et al.’s [46] study in Toronto, Ontario, and DeVetten et al.’s [42] study in Calgary, Alberta, though both tested for parasitic infections, the order and purpose used for the microscopy and enzyme immunoassay were different. Müller et al. [46] followed Public Health Ontario’s recommendations [78] by using microscopy first, followed by enzyme immunoassay (EIA) to differentiate or confirm the Entamoeba species. In contrast, [42] prioritized EIA or multiplex PCR for initial screening, with microscopy as a secondary method, consistent with Alberta Health Service protocols [79]. Such variability, while not unique to refugee populations, has greater implications for them. Refugee children may present with infections uncommon in the general Canadian population, such as tropical parasites or vaccine-preventable diseases. Inconsistent diagnostic sensitivity across measurements can lead to missed or delayed diagnoses [80], emphasizing the need for Canadian standardized protocols for refugee health screening.

In contrast, the measurement practices of non-communicable diseases were more consistent with minor variations. Most studies used body mass index and growth charts from the World Health Organization (WHO) or CDC to assess height and weight. However, more advanced assessments, such as dual-energy X-ray absorptiometry (DXA), revealed gaps in normative data, particularly for children under the age of eight. The International Society for Clinical Densitometry [81] notes the absence of validated reference standards for children under three, limiting the interpretability of DXA results in younger age groups. While this limitation is not exclusive to refugee children, it is especially problematic in this population, where growth delays and nutritional deficiencies are more prevalent and early intervention is critical.

For assessment of development and mental health in young children, tools like the Ages and Stages Questionnaire, the Parents’ Evaluation of Developmental Status, and the Rourke Baby Record are recommended by the Canadian Pediatrics Society and American Academy of Pediatrics; their applications vary, and age ranges are often unspecified. Moreover, few studies have used multi-informant or culturally informed tools, despite evidence that refugee children may express anxiety and depression differently due to trauma and cultural norms [82]. Bernhardt et al. [82] found that refugee children exhibited lower levels of social-interactive play and higher instances of traumatic reenactment play, behaviors linked to parental distress, and adverse experiences. These findings emphasize the need for developmentally appropriate, culturally safe, and trauma-informed assessments. However, the literature remains sparse, and there is no clear gold standard for evaluating mental health in refugee children, particularly in early childhood.

Measurement variability, from the findings, is typical across Canadian pediatric care, not just in refugee contexts. However, the consequences of the variability are amplified in refugees due to their complex health profiles and increased vulnerability. This scoping review findings also extend existing knowledge by revealing how systemic inconsistencies intersect with cultural and developmental complexities, shaping the health experiences and outcomes of refugee children in Canada. While some measurement approaches are well-established, others lack age-appropriate norms, cultural adaptability, or diagnostic sensitivity. What remains unknown is how these inconsistencies affect clinical outcomes, service access, and long-term health trajectories for refugee children, which we propose future research could focus on.

Measurement practices go beyond the technical complexity and require close connection to health equity. When measurement tools and approaches are not developmentally and culturally appropriate, they risk erasing the health needs of refugee children. Consequently, inequities are produced by both the barriers to care and the very systems that define, measure, and document them. Standardization alone will not address these inequities. Rather, a culturally safe measurement approach that recognizes children’s lived experiences, family context, and migration journeys is also key.

### 4.3. Methodological Challenges and Intersectional Considerations

This review reveals substantial methodological gaps and challenges in the literature. While a wide range of physical, developmental, and mental health conditions and outcomes were identified, the depth, consistency, and cultural relevance of measurement practices remain limited. These gaps have significant implications for clinical care, research, and policy development. They also have equity implications as well. When certain health conditions are not routinely and adequately measured, they are effectively rendered invisible within the healthcare system. Invisibility also contributes to inequities, as health conditions that are not seen are not prioritized in planning or funding, reinforcing structural disadvantages for an already vulnerable population.

A key limitation across studies was the inconsistent and often retrospective nature of the data collection. Most studies relied on chart reviews, which introduced missing or incomplete data, particularly affecting pediatric-specific insights. The lack of prospective protocols and longitudinal follow-up limits our ability to assess the long-term health impacts of early conditions, especially communicable diseases. For example, the absence of a formal Canadian screening and management program for latent tuberculosis infection (LTBI) has led to inconsistent practices across provinces, hindering data comparability and standardization of refugee-specific subgroups [83]. Similarly, while adequate measures exist for assessing non-communicable diseases, such as growth and vitamin D levels, the lack of longitudinal, culturally contextualized data prevents meaningful monitoring of refugee children’s health over time. The exclusion of qualitative data further constrained the ability to assess nuanced contextual factors influencing treatment uptake and completion in this population.

Research and practice on the measurement of health conditions and outcomes reported in the literature are heavily skewed toward communicable and non-communicable diseases, despite the known prevalence of developmental and mental health conditions and outcomes in refugee children. Moreover, the review highlights the lack of disaggregated data on refugee-specific subgroups, such as gender, age, or ethnicity. For example, although several studies have noted the vulnerability of ethnic minority girls to vitamin D deficiency, current measurement frameworks do not account for gender or ethnic disparities in bone health outcomes. Similarly, oral and vision health, which are critical components of child well-being, are rarely studied, and no standardized Canadian data exists for newly arrived refugee children.

In addition to the limited research on development and mental health outcomes, this review also revealed challenges in measuring developmental and mental health conditions and outcomes among refugee children. Many screening tools rely heavily on parent-reported data, which can be influenced by cultural norms, perceptions, and expectations, potentially affecting the accuracy and appropriateness of assessments [84,85]. Additionally, these cultural differences have implications for how developmental conditions and outcomes are recognized as well as how screening tools function across diverse populations–the measurement invariance of the tools. Tools such as the Parents’ Evaluation of Developmental Status, which have been translated into other languages, for example, may suffer from measurement invariance, compromising their validity and reliability [86]. As a result, culturally nuanced developmental conditions or outcomes often go undetected. Language barriers and low health literacy further complicate the interpretation of developmental screening results, often leading to underdiagnosis or delayed identification of conditions. These challenges are compounded by the lack of comprehensive epidemiological data on developmental disabilities and delays among refugee children in Canada. Existing data are limited and based on small, non-representative samples, making it difficult to understand the true scope of need.

Mental health measurements were similarly fragmented. The lack of standardized Canadian screening protocols for mental health conditions and outcomes in newly arrived refugee children in Canada contributes to variability in diagnostic practices and potential underreporting. Our findings align with the findings of [32] on the cross-cultural invalidity of screening and measurement tools and the lack of consensus on theoretical frameworks or constructs for measuring trauma and mental health, especially among young refugee children. Furthermore, most studies have relied on symptom-based diagnoses during early resettlement, which can obscure chronic or trauma-related conditions. Moreover, the use of Western diagnostic frameworks cannot fully capture culturally specific expressions of mental health. Few tools have been validated for use with immigrants, refugees, and asylum seekers. This finding is in line with the Canadian Collaboration for Immigrant and Refugee Health, for example, which recommends against routine post-traumatic stress disorder (PTSD) screening due to the potential for harm and lack of validated tools [7].

These findings demonstrate that culturally safe care goes beyond respectful clinical encounters; it also requires culturally safe measurement. Screening tools that are not validated for refugee populations risk misinterpreting or medicalizing normal cultural expressions or missing them entirely. As a result, refugee families may be further marginalized by the systems intended to support them. A shift toward culturally safe measurement would involve co-designing assessment tools with the community and recognizing culturally situated expressions of play, attachment, distress, and resilience.

### 4.4. Implications for Practice, Research, and Policy

This scoping review offers novel insight into health measurement approaches for refugee-serving PHCs, with implications for how research is conducted and how policies can guide healthcare. A key learning from this review is that the current literature pays limited attention to social determinants of health, despite strong evidence that these factors significantly influence refugee child health outcomes. Although refugee children and their families face compounded barriers, they are often underrepresented in existing data systems [87]. Their omission limits their ability to contextualize health conditions and outcomes within the lived realities of refugee families. To fully understand refugee child health conditions and outcomes, future research must adopt a system perspective that goes beyond biomedical indicators to include ecological factors at the micro, meso, and macro levels and how they interact to impact a child’s health outcomes [88,89,90,91]. This system perspective includes cultural preferences, family and community involvement, migration-specific experiences, and collaborative decision-making [88,92]. Furthermore, mixed-methods research that integrates both qualitative and quantitative data is essential to capture the complex interactions of the factors and to inform more equitable and contextually relevant health interventions.

#### 4.4.1. Practice

The findings from the review pointing to evidence of many inconsistencies suggest the need to standardize and strengthen health measurement approaches in refugee-serving PHCs. Providers should adopt evidence-based tools, such as the Rourke Baby Record and the Ages and Stages Questionnaire, which are evidence-based and already widely used in Canadian pediatric care. These tools provide a strong foundation for implementing consistent, age-appropriate, and culturally safe assessments for refugee children. To further improve diagnostic accuracy and support early intervention, the Canadian healthcare system should implement prospective data collection protocols. Standardized data practices would enhance the ability to improve the accuracy of diagnoses, measure health outcomes, allocate resources effectively, and tailor services to the unique needs of refugee populations. By doing so, providers can deliver more equitable, responsive, and high-quality care to refugee children and their families.

With Canada being one of several countries with active refugee resettlement programs, there is a very strong value for international coordination on data collection, measurement, and reporting. Collaboration with other resettlement countries and international entities such as the WHO and UNHCR could support more comprehensive and standardized data on refugee children’s health conditions and outcomes. A broader coordinated system could incorporate shared definitions, standardized measurement tools, and consistent reporting mechanisms to improve comparability across jurisdictions.

Such a system could include core indicators for monitoring refugee children’s health conditions and outcomes, and relevant contextual variables related to their migration pathways, family composition, and post-arrival social determinants of health. Ultimately, such a system will strengthen early identification, support surveillance, align with clinical guidelines, and ensure more equitable resource allocation.

#### 4.4.2. Research

Future research should prioritize the development and validation of culturally and linguistically safe tools tailored to refugee populations. Future studies must employ longitudinal and mixed-methods designs to measure how early health conditions and measurement variability affect long-term outcomes. To improve transparency and comparability, researchers should report age ranges, reference standards, and tool selection criteria. Additionally, integrating qualitative methods to capture the lived experiences, health behaviours, and care-seeking patterns of refugee families provides insights that are essential for designing responsive and inclusive health systems.

#### 4.4.3. Policy

The study findings revealed critical systemic gaps that hinder equitable health service delivery in Canada. Policymakers must address systemic gaps by coordinating national efforts to standardize screening and assessment protocols, especially for developmental and mental health conditions. Current provincial inconsistencies in diagnostic criteria and data collection practices undermine care quality and equity. Federal leadership should coordinate the development and enforcement of culturally appropriate measurement protocols and equip providers with the relevant training and resources needed for effective implementation. Strengthening Canadian data systems to capture disaggregated, refugee-specific health conditions or outcomes will enable more targeted, evidence-based policy decisions and promote health equity across jurisdictions.

### 4.5. Limitations

This study has several limitations. First, the majority of the included studies employed retrospective or cross-sectional designs, limiting our ability to draw causal inferences or assess long-term health outcomes. Additionally, we thematically categorized the findings through inductive analysis. Future studies may use conceptual frameworks to guide their analysis, including, but not limited to, health equity and culturally safe frameworks. Second, most studies relied on chart reviews and electronic medical records, which can be incomplete or inconsistently documented, particularly for pediatric-specific data. Third, the quality of the included studies was not formally assessed, which may have affected the conclusions drawn. Additionally, the underrepresentation of qualitative studies limits the understanding of contextual and cultural factors influencing health outcomes and care-seeking behaviours. There was also notable variability in measurement tools used across studies, compounded by the absence of standardized Canadian protocols for refugee child health measurement. Lastly, the strict focus on studies on health conditions and outcomes of refugee children aged from birth to 5 and the measurement of these conditions and outcomes limited the pool of available studies. Future research could focus on conducting longitudinal and mixed-methods studies to better understand the long-term impacts of resettlement on physical, mental, and developmental health.

## 5. Conclusions

This review highlights the disproportionate focus on physical health, particularly non-communicable diseases, and reveals significant gaps and variability in measurement approaches for young refugee children. While the measurement challenges identified in this review are not unique to refugee children, their impact is more pronounced in this vulnerable population. The findings extend the existing knowledge on gaps in the measurement of child health conditions and outcomes. It also reveals how systemic inconsistencies intersect with cultural and developmental complexities, shaping the health experiences of young refugee children in Canada. Addressing these gaps through coordinated clinical and policy efforts ensures that all children, regardless of origin, receive accurate, timely, and equitable care in Canada. Together, this review also highlights a conceptual shift–improving refugee children’s health in Canada is not simply expanding healthcare services but advancing health equity through a culturally safe system of measurement. Measurement is a gatekeeper of visibility, access, and resources; as such, what is not measured accurately leads to poor clinical decision-making and weak policy responses, further perpetuating inequities.

## Figures and Tables

**Table 1:** Inclusion and exclusion criteria based on the PICO framework.

Criteria	Inclusion (Study Meets All Criteria)	Exclusion (Study Meets Any Criteria)
Population	Focus on refugees, asylum seekers, or displaced persons	Immigrants, adolescents, youth and adults, newcomers
Age	A sample of refugee children from birth to 5 years old in the population	Adolescents, youth and adults
AND		
Setting (Context)	Refugee-receiving primary healthcare clinic/centre providing services to refugee/immigrant	
AND		
Phenomena of Interest (Concept)	Health conditions or outcomes of young children and/or health conditions or outcomes measurement techniques and tools	Focus on acute/emergency health issues
Study design/types of publication	Quantitative, qualitative, mixed-methods studies, interventional studies/RCTs, review articles, secondary data analysis, grey literature, conference proceedings, preprints, and research letter	Opinion papers or commentaries or pieces, books, presentations, conference abstracts, case studies
Results of studies	Results presented for Canadian children < 6 years. Health outcomes must be disaggregated for this population but not health measurements	
AND		
Country	Canada	Any study that does not include Canada
Language	English	Published in any language other than English

**Table 2:** Summary of results at a glance.

	Refugee Child Health Conditions and Outcomes in Canada Identified in Papers Included in Our Study	Measurement of Refugee Child Health Conditions and Outcomes by Refugee-Serving PHCs
Communicable Diseases	Gastrointestinal infections (e.g., Giardiasis, Cryptosporidiosis) Parasitic infections (e.g., worms, Strongyloides stercoralis, and Schistosoma) Hepatitis B Tuberculosis and latent tuberculosis infections (LTBIs)	O&P—Microscopic examination and enzyme immunoassay Hepatitis B—Surface antigen (HBsAg) and core antibody (anti-HBc) Varicella—Immunoglobulin (IgG) positive test Latent Tuberculosis—Interferon-Gamma Release Assay (IGRA) and Tuberculin Skin Test
Non-communicable Diseases	Malnutrition and bone density Haematological conditions Immunization status Oral health Vitamin deficiencies (e.g., A, B, and D)	Growth/Malnutrition—Birth weight (Very low (400–1499 g), low (1500–2499 g), and normal (32,500 g) Bone Density—DXA (body composition, bone mineral content, and density of total body, hip, and lumbar spine) Vitamin D—serum 25-hydroxyvitamin D (25[OH]D); (≥50 nmol/L or ≥75 nmol/L) Anaemia—Hemoglobin levels (110 g/L; children aged 6–59 months) Complete Immunization—three doses of Diphtheria/Pertussis/Tetanus/Polio/Haemophilus influenzae type B (DPTP/Hib) with a booster and one dose of measles, mumps, and rubella vaccine. Diabetes—random capillary blood glucose test (≥7.8 mmol/L), fasting glucose (≥6.1 mmol/L or ≥7.0 mmol/L), random glucose (≥11.1 mmol/L), HbA1c (≥6.0% or ≥6.5%) Oral Health—Dental caries (dmft/DMFT); Plaque accumulation (Simplified Oral Hygiene Index) Eye Health—Visual screening, slit-lamp examinations, direct dilated fundoscopy, and refractive index measurements.
Developmental and Mental Health of Refugee Children	Neurodevelopmental delays and disabilities Behavioural difficulties Emotion dysregulation, aggression, anxiety, and depression	Developmental Delays—Enhanced 18-month well-child visit (EWCV); Nipissing District Assessment tool; Rourke Baby Record; Parents Evaluation of Developmental Status; Ages and Stages Questionnaire, Autism—Autism Diagnostic Observation Schedule, Modified Checklist for autism in toddlers, and Childhood Autism Rating Scale screening tools. Mental Health Condition—2014 Ontario Child Health Study Emotional Behavioural Scales, Diagnostic and Statistical Manual of Mental Disorders-5, International Classification of Diseases (ICD-9 and ICD-10)

## Data Availability

The original contributions presented in this study are included in the article/Supplementary Material. Further inquiries can be directed to the corresponding author(s).

## Supplementary Materials

The following supporting information can be downloaded at: https://www.mdpi.com/article/10.3390/ijerph23010092/s1, Supplementary File S1: PRISMA 2020 Check list.

## Abbreviations

The following abbreviations are used in this manuscript:

ADHD	Attention Deficit/Hyperactivity Disorder
ASD	Autism Spectrum Disorder
CDC	Centre for Disease Control
CINAHL	Cumulative Index to Nursing and Allied Health Literature
CMAJ	Canadian Medical Association Journal
DPTP	Diphtheria/Pertussis/Tetanus/Polio
DXA	Dual-Energy X-ray Absorptiometry
EIA	Enzyme Immunoassay
EMR	Electronic Medical Record
GARs	Government-Assisted Refugees
Hib	Haemophilus influenzae type B
ICD-10-CA	International Classification of Diseases and Related Health Problems, the 10th Revision, Canada
IGRA	Interferon-Gamma Release Assay
IgG	Immunoglobulin G
LTBI	Latent Tuberculosis Infection
NCHC	New Canadians Health Centre
O&P	Stool Ova and Parasites
PHC	Primary Health Centre/Clinic
PICO	Patient, Intervention Comparison and Outcome
PRISMA-ScR	Preferred Reporting Items for Systematic Reviews and Meta-Analyses for Scoping Reviews
PSRs	Privately Sponsored Refugees
REC	Research and Evaluation Committee
WHO	World Health Organization

## Appendix A

Box A1 Full search string.

1 Refugees/or (refugee* or asylum seeker* or asylee* or “displaced person*” or (claimant* and asylum)).mp.
2 (refugee clinic* or asylum clinic or refugee health program* or asylum health program*).mp. or Community Health Centers/or Community Mental Health Services/or Community health services/or (community networks/and (“health service*” or “health care” or healthcare or “primary care” or “migrant health” or “primary health*” or “public health”).mp.) or ((“community health” or “neighborhood health” or “primary health” or “primary care” or “public health” or “migrant health” or “refugee health” or “asylum seeker health” or “asylum health” or “claimant* health”) adj3 (center* or centre* or clinic? or service* or network*)).mp. or ((health or healthcare) adj3 (center* or centre* or clinic? or service*)).mp. or ((“community health” or “neighborhood health” or “primary health” or “primary care” or “public health”) adj5 (refugee* or asylum seeker*)).mp.
3 Child/or exp infant/or exp pediatrics/or (pediatric* or paediatric* or newborn* or congenital* or infan* or baby or babies or neonat* or pre-term or preterm* or premature birth* or child* or preschool* or pre-school* or kindergarten* or kindergarden* or nursery school* or ((day care* or daycare*) not adult*) or toddler* or boy or boys or girl* or grade-1* or grade-one* or m*-old* or 1-y*-old* or one-y*-old* or 2-y*-old* or two-y*-old* or 3-y*-old* or three-y*-old* or 4-y*-old* or four-y*-old* or 5-y*-old* or five-y*-old*).mp.
4 (“health status” or “nutritional status” or “vaccination status” or “immunization status” or “health screening” or “disease screening” or “Health outcome*” or “outcome measure*” or PROM or PROMs or “health needs” or “health concerns” or “health issues” or “health conditions” or “healthcare access” or “healthcare utilization” or (health* adj (need* or require*))).mp.
5 (malnutrition* or bone density* or health or “mental health” or anxiety or depress* or ptsd or trauma* or posttraumatic or post-trauma* or autis* or ADHD or “attention deficit” or “neurodevelpmental disorder*” or anemia or “iron deficienc*” or “vitamin deficienc*” or “infectious diseases” or “intestinal infection*” or “malaria” or parasite* or “upper respiratory infection*” or pneumonia or measles or mumps or rubella or “whooping cough” or hepatitis or polio* or diptheria or pertussis or varicella or tetanus or HIV or “HIV/AIDS” or tuberculosis or “human immunodeficiency virus” or obesity or overweight or underweight or wasting or “dental health” or “oral health” or “gum disease*” or “dental caries” or “blood elevated levels” or rickets or “stunt* growth”).mp.
6 (Vitamin or iron or lead or calcium).mp.
7 (“Child development indicators” or ((child* or infant* or neonat* or toddler*) adj4 milestone)).mp.
8 4 or 5 or 6
9 1 and 2 and 3 and 8
10 (Canad* or British Columbia* or Alberta* or Saskatchewan or Manitoba* or Yukon or Northwest Territories or NWT or Nunavut or Ontario or Quebec* or Nova Scotia or Prince Edward Island or Newfoundland or Labrador or New Brunswick or Toronto or Vancouver or Montreal or Edmonton or Calgary or Winnipeg or Ottawa or Halifax).mp,in.
11 9 and 10

## Appendix B

**Table A1:** Summary of population characteristics of papers included in the review (*n* = 25).

Study ID	Study Aim	Study Design	Province	Population Age	Country/Region of Origin
[33]	A systematic review that describes the extent, quality and cultural appropriateness of current research on the health conditions of refugee children aged 0–6 years settled in high-income countries	Systematic review	Not Canadian specific, but includes papers in Canada	0–6	High-income countries
[41]	Characterize the demographics and health status of North Korean refugees who have accessed care at a refugee clinic in Toronto	Retrospective chart review; cross-sectional, other: Community-Based Participatory Research (CBPR) methodology	Ontario	All	North Korea; Swaziland; Saudi Arabia; Croatia; Iran; Afghanistan; Eritrea; Ethiopia; Nigeria; North Korea; Hungary; Other
[42]	To determine the prevalence of intestinal parasites and rates of stool testing compliance, as well as associated patient characteristics	Retrospective chart review, cross-sectional	Alberta	<6 years; 6–18 years; 19–39 years and 40 years	Sub-Saharan Africa; North Africa; Middle East; Asia; Latin America; Europe or North America
[43]	To determine the level of serum 25-hydroxyvitamin D (25-(OH) D) in the paediatric refugee population residing in Sherbrooke, Quebec, Canada, and to determine variables predicting vitamin D levels including age, sex, BMI, influence of season, ethnicity, previous country of residence and duration of stay in Canada from time of arrival.	Cross-sectional study; retrospective chart review	Quebec	<3 years; 3–<6 years; 6–<12 years; 12 years	Afghanistan; Iraq; Bhutan, Columbia, Guatemala; and Central African Republic
[44]	To describe the population of Syrian refugees who received care at temporary triage clinics, the health issues addressed, and the health services used in the clinics	Non-randomized experimental study; cross-sectional study; retrospective chart review	Ontario	1 month–62 years (55% children)	Syria
[45]	To determine the prevalence of selected infectious diseases among newly arrived refugee patients and whether there is variation by key demographic factors	Retrospective chart review, cross-sectional	Ontario	0–4 years; 5–14 years; 15–24 years; 25–34 years; 35–44 years; 45–54 years; 55–64 years; 65+ years	Africa; Americas; Asia; Eastern Mediterranean; Europe
[46]	This study aims to investigate the prevalence and species of intestinal parasites identified in stool ova and parasite (O&P) specimens in a sample of newly arrived refugees in Toronto, Canada.	Retrospective chart review, cross-sectional	Ontario	0–9 years; 10–19 year; 20–29 years; 30–39 years; 40–49 year; 50–59 years; 60–69 years; 70+	East Asia & Pacific; Europe & Central Asia; Latin America & Caribbean; Middle East & North Africa; North America; South Asia; and Sub-Saharan Africa
[47]	To determine the prevalence of selected chronic diseases among newly arrived refugee patients and explore associations with key demographic factors	Retrospective chart review, cross-sectional	Ontario	0–4 years; 5–14 years; 15–24 years; 25–34 years; 35–44 years; 45–54 years; 55–64 years; 65+ years	Africa; Americas; Asia; Eastern Mediterranean; Europe; North America
[48]	To describe selected anthropometric and health status variables among immigrant and refugee children 6 years of age within an inner city clinic in Toronto, Ontario	Cross-sectional; Retrospective chart review	Ontario	0–6 years	Afghanistan; Myanmar; and Columbia
[49]	To investigate access to effective primary health care services in children of new immigrants to Canada by assessing immunization coverage at age 2	Longitudinal (cohort study)	Ontario	17–24 months	Latin/Central America, Western Europe, North America; Eastern Europe; Middle East; Africa; Southeast and Northeast Asia, Oceania; South Asia
[50]	To measure LTBI treatment acceptance and completion outcomes of LTBI treatment at the REACH clinic in Saskatoon	Retrospective chart review, cross-sectional	Saskatchewan	6 months or older	Africa; Eastern Europe, Mediterranean; Southeast Asia
[51]	To assess the ocular health status of Syrian pediatric refugees in Canada and report the prevalence of vision impairment within this population	Multi-methods cross-sectional study,	Ontario	<1 year; 1–3 years; 4–6 years; 7–9; 10–12; 13–15; 15–18	Syria
[52]	To evaluate factors associated with uptake of a financial incentive for developmental screening at an enhanced 18-month well-child visit (EWCV) in Ontario, Canada.	Cross-sectional study; retrospective chart review	Ontario	17–24 months.	Not specified
[53]	To assess growth indicators for resettled Yazidi and non-Yazidi pediatric refugees from Syria and Iraq.	Retrospective chart review cohort study	Alberta	<5 years and 5 years or older	Iraq and Syria
[54]	To determine whether refugee or asylum-seeking women or their infants experience a greater number or a different distribution of professionally-identified health concerns after birth than immigrant or Canadian-born women.	Quantitative research; longitudinal (cohort study), Retrospective study	Ontario and Quebec	Infants	Refugees: Africa; Asia; Europe; Latin America Asylum-Seekers: Africa; Asia; Europe; Latin America Immigrants Africa; Asia; Europe; Latin America; Northern America
[55]	To determine the 25-hydroxyvitamin D (25[OH]D) serum levels in refugee women of childbearing age and in refugee children; to compare their 25(OH)D levels with the recommended levels in order to determine the prevalence of deficiency; to compare their 25(OH)D levels with those in the general Canadian population in the appropriate age and sex groups; and to investigate the association of vitamin D deficiency with potential risk factors	Cross-sectional study; retrospective chart review	Alberta	0–5 years; 6–11 years; 12–19 years; 20–39 years; 40–45 years	Africa; Asia; Middle East; South America; Other
[56]	To conduct a detailed retrospective cohort investigation of the sociodemographic characteristics, health conditions, and clinic utilization patterns of Afghan refugee patients resettled in Calgary, Canada between 2011 and 2020	Retrospective chart review; other: community-engaged	Alberta	0–4 years; 5–11 years; 12–17 years	Afghanistan
[57]	Explore the health and nutritional status of immigrant and refugee children in Canada, identifying chronic disease risks and health inequities influenced by socioeconomic and lifestyle factors, to inform public health interventions for newcomer families.	Retrospective, cross-sectional study; mixed-methods research		3–13 years	
[58]	To assess the oral health status of refugee children in comparison with that of Canadian children and investigate the extent to which demographic factors are associated with caries experience in this population	Retrospective chart review	Quebec	1–14 years	Africa; Latin America; North America; Middle East; Europe; and Asia
[59]	To identify the risk determinants of caries and record oral hygiene status in recent immigrant and refugee children	Cross-sectional	Saskatchewan	Refugee children: 3–6 years; 6–14 years; 14–16 years Immigrant children: 3–6 years; 6–14 years; 14–16 years	Indian subcontinent, Asia, and rest of the world
[60]	To provide a framework for primary care providers to approach developmental disabilities in both refugee and nonrefugee immigrant populations	Review	NA	NA	NA
[61]	To examine the variation among ethnic populations in the prevalence of anemia, vitamin D and B12 deficiencies among refugee children. The study aims to determine the frequency and distribution of these deficiencies among refugee children, stratified by country of origin and age group	Cross-sectional study; retrospective chart review	Ontario	0–4 year; 5–11 years; 12–16 years	Iraq; Somalia; Myanmar; Afghanistan; Other
[64]	To examine differences in mental health-related service contacts between immigrant, refugee, racial and ethnic minoritized children and youth, and the extent to which social, and economic characteristics account for group differences	Other: retrospective correlational analysts (cross-sectional)	Ontario	4–17 yrs	NA
[71]	Estimate the administrative data-derived diagnostic prevalence of mental disorders (conduct, attention-deficit/hyperactivity disorder [ADHD], and mood/anxiety) for refugee, immigrant, and nonimmigrant children and youth in British Columbia, Canada.	Retrospective chart review, cross-sectional	British Columbia	0–19 years	NA
[62]	To characterize the health and nutritional status of immigrant and refugee children in Canada, specifically focusing on their bone mineral content and vitamin D status	Cross-sectional; mixed-methods research	Saskatchewan	3–4 years; 5–7 years; 8–10 years; 11–13 years	Immigrants: Middle East (e.g., Iran, Iraq, Pakistan); Asia (e.g., Burma, India, Philippines); Africa; Latin America; Eastern Europe; Western Europe and United States Refugees: Middle East (e.g., Iran, Iraq, Pakistan); Asia (e.g., Burma, India, Philippines); Africa; Latin America; Eastern Europe

**Table A2:** Summary of papers included in review (*n* = 25).

Study ID	Outcomes			Measurement Approach Defined
	Communicable	Non-Communicable	Developmental and Mental Health	
[33]	X	X	X	NA
[41]	X			Stool ova and parasite test—All specimens were first screened for Giardia lamblia and Cyptospokidium using an enzyme immunoassay, then positives were confirmed with direct fluorescent antibody testing.
[42]		X		Laboratory measurements of 25-(OH)D levels determined by Elecsys Vitamin D Total Assay.
[43]	X	X	X	NA
[44]	X			HIV serology, hepatitis B serology (surface antigen and surface antibody), hepatitis C antibody, Strongyloides serology, Schistosoma serology, stool ova and parasites, gonorrhea and chlamydia testing (culture or nucleic acid amplification test), syphilis testing, and varicella immune status (varicella immunoglobulin G antibody)
[45]	X	X		BMI, WHO Anemia guidelines, height, weight
[46]		X		Haemoglobin levels and diabetes screening test results (fasting blood glucose, random blood glucose, or hemoglobin A1c [HbA1c] measurement)
[47]	X	X		Height and weight abnormalities, determined by the WHO Child Growth Standards (using a cut-off z-score of ≤−2, which indicates a percentile measurement of approximately ≤2.3). Data were also collected regarding the presence of iron deficiency (Defined as ferritin levels below the age-specific laboratory lower reference limits); anemia (defined as hemoglobin levels below the age-specific laboratory lower reference limits); parasitic enteric infections (positive stool culture for ova and parasites); hepatitis B (positive hepatitis B surface antigen levels); HIV (positive ELISA test); and elevated lead levels (lead levels ≥ 0.48 μmol/L).
[48]		X		Immunization rates (categorized as 85%, 90%, or >95% complete immunizations) and birth weight
[49]	X			QuantiFERON-TB Gold Plus 4-tube assay (IGRA) and Mantoux method (TST)
[50]		X		Visual screening, slit-lamp examination, direct dilated fundoscopy, and refractive index measurements.
[51]		X		5 immunizations given after age 7 weeks as being up to date, representing the recommended 3 doses and 1booster of Diphtheria/Pertussis/Tetanus/Polio/ Haemophilus influenzae type B (DPTP/Hib) given at 2, 4, 6, and 18 months, and 1 combined dose of measles, mumps, and rubella given after age 12 months.
[52]		X		Height and Weight
[53]		X	X	Project nurse assessment post-birth, 7–10 days and four months. Assessed for the presence of health concerns based on a list developed from standards for postpartum care. Nipissing Assessment Tool Vitamin D supplements Infant weight
[54]		X		Laboratory measurement using the standard DiaSorin Liaison 25(OH)D vitamin D assay.
[55]	X	X	X	Based on the ICD-10
[56]		X		Physical exam: Dual-energy X-ray absorptiometry (DXA). Blood sample for serum vitamin D analysis. Questionnaire: Modified version of the Canadian Community Health Survey (CCHS) 2008 socio-economic and demographic questionnaire, the CCHS Food security Questionnaire, Statistics Canada’s Children’s Physical Activity questionnaire, and serial 24 h dietary recalls.
[57]		X		Dental caries diagnosis (dmft/DMFT) for primary and permanent teeth. Oral hygiene was scored using the simplified oral hygiene index. Gingival health status was measured visually without probing. Patients’ malocclusion assessment looked at crossbite, open bite, overbite, and overjet.
[58]		X		Clinical Examinations–Assessment of the presence of the number of decayed, missing and filled teeth (dmft/DMFT). Oral hygiene status evaluated by Simplified Oral Hygiene Index (OHIS).
[59]			X	NA
[60]		X		“Hemoglobin Levels: Hemoglobin levels were measured to evaluate anemia among refugee children. The cut-off levels for anemia were based on age and sex-specific guidelines from the World Health Organization (WHO). Vitamin D Levels: Serum 25(OH)D levels were measured to assess vitamin D status. Vitamin D is crucial for bone health, immune function, and overall physical development. Vitamin B12 Levels: Serum vitamin B12 levels were measured to determine the prevalence of vitamin B12”
[61]			X	Questionnaire tool: 2014 Ontario Child Health Study (OCHS)
[64]			X	Identified Indicators using symptoms
[71]		X		Physical exam: Dual-energy X-ray absorptiometry (DXA). Blood sample for serum vitamin D analysis. Questionnaire: Modified version of the Canadian Community Health Survey (CCHS) 2008 socio-economic and demographic questionnaire, the CCHS Food security Questionnaire, Statistics Canada’s Children’s Physical Activity questionnaire, and serial 24 h dietary recalls. Blood sample to assess serum glucose, total cholesterol, and serum vitamin D. Anthropometric measurements including height, weight, and waist circumference

Note: X denote the included study reported on the outcome. NA denote measurement approach not defined in the included study.
